# Facile Preparation and Highly Efficient Catalytic Performances of Pd-Cu Bimetallic Catalyst Synthesized via Seed-Mediated Method

**DOI:** 10.3390/nano10010006

**Published:** 2019-12-18

**Authors:** Fangke Zhan, Juanjuan Yin, Jingxin Zhou, Tifeng Jiao, Lexin Zhang, Meirong Xia, Zhenhua Bai, Qiuming Peng

**Affiliations:** 1 State Key Laboratory of Metastable Materials Science and Technology, Yanshan University, Qinhuangdao 066004, China; kekelittlebear@126.com (F.Z.); pengqiuming@ysu.edu.cn (Q.P.); 2 Hebei Key Laboratory of Applied Chemistry, School of Environmental and Chemical Engineering, Yanshan University, Qinhuangdao 066004, China; jjy1729@163.com (J.Y.); zhanglexin@ysu.edu.cn (L.Z.); xmr0125@126.com (M.X.); 3 National Engineering Research Center for Equipment and Technology of Cold Strip Rolling, Yanshan University, Qinhuangdao 066004, China; bai_zhenhua@aliyun.com

**Keywords:** seed-mediated, bimetallic catalyst, nanocomposites, catalyst

## Abstract

With the rapid development of industry, the problem of environmental pollution has become increasingly prominent. Exploring and preparing green, efficient, and low cost catalysts has become the key challenge for scientists. However, some conventional preparation methods are limited by conditions, such as cumbersome operation, high energy consumption, and high pollution. Here, a simple and efficient seed-mediated method was designed and proposed to synthesize a highly efficient bimetallic catalyst for catalyzing nitro compounds. A Pd-Cu bimetallic composite (BCM) can be prepared by synthesizing the original seed crystal of precious metal palladium, then growing the mature nanocrystalline palladium and supporting the transition metal copper. Importantly, after eight consecutive catalytic cycles, the conversion of the catalyzed 2-NA was 84%, while the conversion of the catalyzed 4-NP was still 72%. And the catalytic first order rates of 2-NA and 4-NP constants were 0.015 s^−1^, and 0.069 s^−1^, respectively. Therefore, current research of nanocomposites catalyst showed great significance for serious environmental pollution problems and the protection of living environment, providing a new idea for the preparation of new bimetallic catalytic materials.

## 1. Introduction

With the rapid development of modern society, the problem of environmental pollution has become increasingly serious, and the types and quantities of chemical pollutants in the atmosphere and soil have increased rapidly. In particular, any discharge of chemical dyes poses a serious threat to human health. In recent years, how to obtain efficient green, low-cost, recyclable catalysts has become a key and difficult point in this field. Some researchers have developed a variety of different methods to deal with chemical dyes, such as adsorption degradation [1,2], electrocatalytic oxidation [3], biodegradation [4,5] and photocatalytic degradation, etc. [6,7,8].

In addition to the above methods, endless streams of catalyst materials have been developed, such as p-nitrophenol (4-NP) and o-nitroaniline (2-NA) [9,10,11,12,13], in order to solve these compounds which pose a major hazard to the environment and human health. In addition, a common catalyst for palladium is well-known [14,15]. In recent years, some Pd-doped composite catalyst materials have been reported. Palladium has a very good catalytic activity for the coupling reaction of carbon-carbon bonds due to its strong affinity for hydrogen, and its cost advantage is superior to other metals such as platinum. At the same time, palladium can be used in the Suzuki reaction [16,17], purification storage and detection [18,19] and dye catalysis [20,21]. Due to high electrical conductivity and thermal conductivity of transition metals, and because the cost is much lower compared to precious metals, special attention to copper metal has continued to increase [22,23,24,25,26]. Therefore, more and more composite nanomaterials of two metals, palladium and copper, have appeared in recent years. Iwamoto et al. successfully prepared a nitrate-removed nitrate from brine by using an anion exchange resin (AIER) as a carrier [27]. Ye et al. have prepared Pd-Cu nanoparticles supported on reduced graphene oxide nanosheets with high catalytic performance for two-step reduction [28]. Mao et al. designed and obtained well-dispersed Pd-Cu bimetallic nanocrystals, which showed good performance in both ethanol electrooxidation and tire peroxidation [29,30,31,32]. However, the above reports may have cumbersome preparations, adverse effects such as resource consumption and environmental pollution. In this work, a uniform, low-cost, green, non-polluting bead-like palladium particle was prepared by seed-mediated method, which overcomes particle agglomeration due to the large surface energy. After the copper is loaded, the shape can still be clearly controlled. Other than that, synthesizing structurally defined bimetallic NPs has been a great challenge for a long time. But the seed-mediated method in this study used uniform Pd seed nanocrystals as the cores when growth Pd nanocrystals, excluded large-scale heteronuclear crystals generated and spontaneous nucleation firstly. Secondly, the second metal is loaded on the premise that the Pd nanocrystals are still highly homogeneous. Compared with the one-step methodology, the size distribution of the particles is smaller, and the preparation process is simpler and easier to control [33]. The novel Pd-Cu bimetallic composite (BCM) catalyst prepared by the method has excellent catalytic degradation performance for p-nitrophenol (4-NP) and o-nitroaniline (2-NA). The current research work provides a broad application prospect for palladium-based bimetallic composite materials in the field of chemical dye catalytic degradation and sewage treatment.

## 2. Materials and Methods

### 2.1. Materials

Ascorbic acid (AA), palladium (II) chloride (PdCl_2_, 59–60%), HCl solution, potassium iodide (KI, 99%) purchased from Aladdin Reagent (Shanghai, China). Copper (II) chloride (CuCl_2_, 98%), and sodium dodecyl benzene sulfonate (SDBS) (90%) were purchased from Sigma Aldrich (St. Louis, MO, USA). Ethanol (C_2_H_5_OH, analytical reagent) was supplied by Tianjin Guangfu Fine Chemical Research Institute (Tianjin, China), 2-nitroaniline (2-NA, 99%), and 4-Nitrophenol (4-NP, 99%) were obtained from Alfa Aesar (Beijing, China). Ultra-pure water was obtained by a Milli-Q Millipore filter system (Millipore Co., Ltd., Bedford, MA, USA) with a resistivity of 18.2 MΩ cm^−1^. All chemicals were used as received without purification.

### 2.2. Fabrication of Pd-Cu Bimetallic Catalyst

The 10mM H_2_PdCl_4_ solution was obtained by adding 0.1773 g of PdCl_2_ in 10 mL of 0.2 M HCl solution and then moved into the volumetric flask before diluting to 100 mL ultra-pure water.

### 2.3. Synthesis of Palladium Seeds

The preparation of palladium was obtained according to the following method. Specifically, 20 mL of 12.5 mM CTAB solution was added to 1 mL of 10 mM H_2_PdCl_4_ solution heated at 95 °C under stirring. After 5 min, as the mixture was mixed into the solution, 160 μL of freshly prepared 100 mM AA solution was added quickly, then the mixture was inverted once, and then to avoid interference, the reaction was allowed to carry on for 30 min. The solution, filled with Pd seed, was stored at 30 °C for future use as seeds.

### 2.4. Seed-Mediated Growth of Bead-Chain Palladium Nanocrystals

In a typical synthesis, 25 μL of 1 mM KI solution was added to 5 mL of 100 mM SDBS solution kept at 40 °C, 80 °C, 90 °C, respectively, 125 μL portion of 10 mM H_2_PdCl_4_ solution and 40 μL of seed palladium solution as synthesized were then added. Finally, 100 μL of freshly prepared 100 mM ascorbic acid (AA) solution was added by drop, finally the solution was mixed thoroughly. The resulting solution was sited in a water bath for 12, 1, 0.5 h corresponding to the reaction temperature. The reactions were stopped by centrifugation (8000 rpm, 10 min). The precipitates was diluted and dispersed with 3 mL of water.

### 2.5. Synthesis of Pd-Cu Nanomaterial

For the part of Pd-Cu nanomaterial growth, 2 mL aqueous SDBS (0.2 M) solution was added to the solution which has mixed 0.025 mL H_2_PdCl_4_ (10 mM) solution and 0.05 mL of CuCl_2_ (10 mM) aqueous solution. What has to be aware of is that the preparation of the CuCl_2_ solution needs a small amount of hydrochloric acid solution to adjust the pH value. Following with 1.5 mL AA (0.1 M) aqueous solution, the 21.4 mL of water was added, and then 1 mL solution containing the Pd nanocrystal was added. The vials was capped, inverted once, and allowed to sit undisturbed in a 25 °C water bath 12 h. The product was collected by centrifugation of the solution at 8000 rpm for 20minutes; the supernatant was removed, and the final products were dispersed in ultrapure water and ethanol for UV-vis extinction spectra characterization. Two more centrifugations (8000 rpm, 10 min) were applied to the final product for XRD characterization, transmission electron microscope (TEM), and scanning electron microscopy (SEM).

### 2.6. Catalytic Performance Test

The 2-NA or 4-NP (2 mL, 5 mM) solution was added to a freshly prepared NaBH_4_ (20 mL, 0.01 M) solution at room temperature. In this case, NaBH_4_ is a reducing agent throughout the reduction reaction. 1 mg of the Pd-Cu bimetallic composite catalyst was added to 2 mL of ethanol to form a stable 0.5 mg/mL suspension. A portion of suspension (2 mL, 0.5 mg/mL) was then added to the 2-NA or 4-NP solution for catalytic reduction, and the whole process was tested at room temperature and under UV spectrum. The Pd-Cu bimetallic composite catalyst was recycled in the next cycle experiment before washed 3 times by the ultrapure water and ethanol. The same solid powder was repeated use along with fresh 2-NA or 4-NP and NaBH_4_ aqueous solutions.

### 2.7. Characterization

The synthesized materials were treated to remove water by freeze drying at a temperature of −48 °C for over 24 h via a FD-1C-50 instrument (Beijing Boyikang Experimental Instrument Co., Ltd., Beijing, China). TEM studies were performed with a transmission electron microscopy (TEM, HT7700, Hitachi high technologies Corporation, Ibaraki, Japan). The structures of Pd-Cu nanomaterials were investigated via scanning electron microscope (SEM) Field Emission Gun FEI QUANTA FEG 250 (FEI Corporate, Hillsboro, OR, USA). X-ray diffraction (XRD) were obtained with an X-ray diffractometer equipped with a Bragg diffraction setup (SMART LAB, Rigaku, Akishima, Japan) and a Cu Kα X-ray radiation source to further characterize the obtained materials. High-resolution transmission electron microscopy (HRTEM, Tecnai-G2 F30 S-TWIN, Philips, Eindhoven, Netherlands) images were acquired with a JEM-2010 electron microscope (Hitachi, Tokyo, Japan) operated at 200 kV. X-ray photoelectron spectroscopy (XPS) was performed using a Bragg diffraction setup (ESCALAB 250Xi XPS) with Al Kα X-ray source.

## 3. Results and Discussion

### 3.1. Structural Characterization of Pd-Cu Nanocomposites

**Figure 1:**
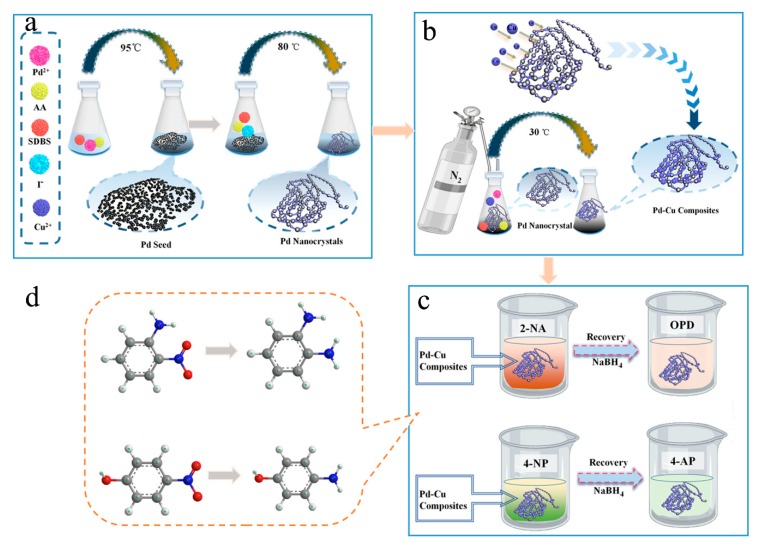
Schematic illustration of preparation as well as catalyt ic application of Pd-Cu bimetallic composite materials (BCM): prepared Pd seed and Pd nanocrystals (**a**); prepared Pd-Cu composites (**b**); catalytic reduction test of Pd-Cu composites (**c**); ball-and-stick molecular model of 2-NA, OPD and 4-NP,4-AP, respectively (**d**).

In this work, Pd-Cu bimetallic composite materials (BCM) synthesized by Seed-mediated synthetic method shows homogeneous appearance and high catalytic performance. First, a solution of H_2_PdCl_4_ (1 mL, 10 mM) was added to SDBS solution (20 mL, 12.5 mM), and then kept the mixture at 95 °C for 5 min. As shown in Figure 1a, as the AA solution was added, the Pd^2+^ ions were reduced to form Pd seed NPs. Through adding Pd seed into growth solution containing I^−^, SDBS and the reducing agent fresh AA solution, Pd atoms begin to deposit on Pd seeds NPs. Bakshi has reported that the correlation between sodium dodecyl sulfate (SDS) and its ability in designing nano-morphologies. Therefore, it can be inferred that the SDBS, which was a kind of anionic surfactant, which is the same as sodium dodecyl sulfate (SDS), was used to form a dense layer. And the dense layer induces the growth of palladium particles on one crystal plane and restricts the growth on other crystal faces, then the bead-chain palladium nanocrystals has been given [32]. And adding I^−^ ions could avoid the structure fluctuations of the palladium nanoparticles and depress the spontaneous nucleation of it. That can be shown in detail by Niu et al. [33]. A set of comparative tests was set with the preparation Pd nanocrystals, and the phenomenon of spontaneous nucleation was obvious with absence of I^−^ [33]. Therefore, it is very important to add halogen elements iodine to generate a complete palladium crystal. Then, the seed-mediated method was also used in the step of palladium nanocrystals composite copper particles as shows in Figure 1b. At this time, palladium nanocrystals were added as seeds to the growth solution containing Cu^2+^ ions, and Cu^2+^ ions were uniformly reduced on the beads of palladium to form the bead chain structure. In order to prevent copper from being oxidized by oxygen in solution and air during the reduction process, the whole process requires nitrogen protection in a slow reduction process. Next, the catalytic activity of Pd-Cu bimetallic composite materials (BCM) for 2-NA and 4-NP was studied as Figure 1c performed. In this part, the catalytic activity for the functional group -NO_2_ is attributed to the mutual synergy of Pd-Cu bimetallic composite materials (BCM). First, when BH_4_^−^ is added to the solution containing 4-NP, electrons are transferred from the BH_4_^−^ donor to the 4-NP receptor, and then amino derivative has been produced. After adding the catalyst, the amino derivative and BH_4_^−^ are adsorbed on the surface of Pd-Cu bimetallic composite materials (BCM) by the combination of hydrogen bonding and physical adsorption. When electrons transfer Pd-Cu NPs, the hydride forms a hydrogen atom and spontaneously undergoes a reduction reaction on the surface of Pd-Cu bimetallic composite materials (BCM). Finally, the prepared 4-nitroaniline (4-AP) was desorbed from the surface of the catalyst [34]. Similarly, the nitro group in the 2-NA molecule should be converted to an amino group under conditions of catalytic hydrogenation (Figure 1d) [35].

**Figure 2:**
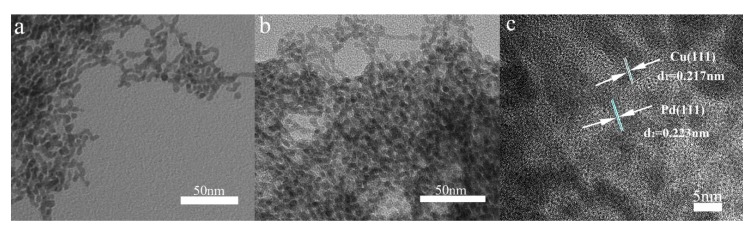
TEM images of pure Pd sample (**a**), Pd-Cu bimetallic composite (BCM) sample (**b**) and HTEM image of Pd-Cu bimetallic composite (**c**).

**Figure 3:**
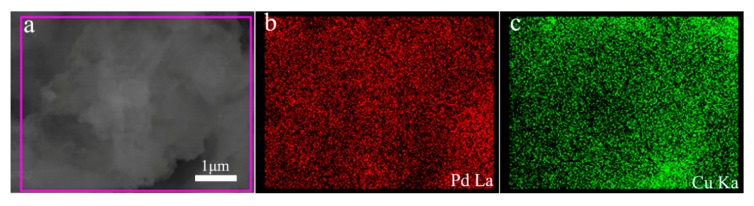
SEM image and of Pd-Cu bimetallic composite nanoparticles (**a**) with Pd/Cu elemental mapping (**b**,**c**).

After adjusting the reaction temperature and the corresponding reaction time, the most suitable products (80 °C, 1 h) are selected, which has the most clear and homogeneous morphology as well as the better performance. To further characterize the morphology of the composite, a TEM test was performed as shown in Figure 2. As can be seen from Figure 2a, the prepared palladium particles are heaped together as many strings of irregular beads. They grow into uniform beads under the action of the growth liquid and the halogen element I^−^ ion, which depress the spontaneous nucleation of Pd atoms when they deposition on the Pd seed nanocrystal [33]. Finally, a uniform bead chain shape is formed. In Figure 2b, the reduced composite of copper causes the diameter of some of the beads on the bead chain to become larger and the color to become significantly darker. In addition, in order to further observe the structure of the prepared Pd-Cu bimetal composite (BCM), high-resolution transmission electron microscopy (HRTEM) analysis is necessary, as shown in Figure 2c. Compared to the TEM image of palladium, the image in Figure 2a clearly shows many beads that loaded the copper atom in the chain are darker than the original pure palladium beads. Obviously, the obtained copper and palladium show a clear crystal structure and a distinct lattice fringe. The plane spacing of the lattice data of copper d(111) is 0.217 nm, while the plane spacing d(111) of the lattice data of palladium is 0.223 nm [36,37]. Importantly, the reported palladium nanocrystals have a clear and distinct morphology, and very uniform dispersion compared to other reports [38,39,40,41]. At the same time, the shape of the bead chain is also very intuitive, and the shape after loading the copper is still very clear. More notably, the palladium and copper molar ratio selected in this report is more suitable for the preparation of homogeneous appearance of Pd-Cu (BCM) compared with other research reports [42]. In addition, as shown in Figure 3, the results of elemental map analysis indicated that the prepared Pd-Cu bimetallic composite materials (BCM) were mainly composed of Pd and Cu elements. These elemental diagrams further suggested that copper was supposed to be reduced on the palladium nanocrystals with the uniform dispersion, which needed to be confirmed by further studies.

**Figure 4:**
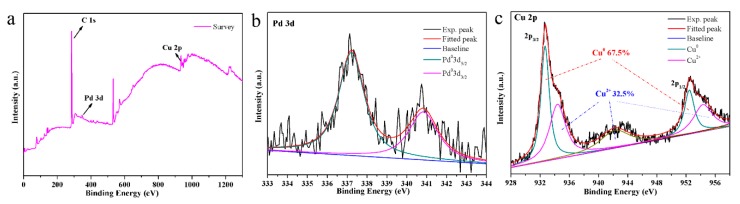
(**a**) wide XPS spectrum of the Pd-Cu bimetallic composite materials (BCM) samples; (**b**) Pd3d peaks; (**c**) Cu2p peaks.

XPS testing of the obtained Pd-Cu bimetallic composite (BCM) for more detailed analysis is of great significance for studying the elemental composition and chemical state of the composite, as shown in Figure 4 [43]. The Pd 3d region shows the ratio of the bimodal binding energy of the spin coupling to the metal Pd in the expected 2:3 by high resolution scanning (Figure 4b). After the composite copper, the content of the main Cu^0^ (67.5%) in the composite material is much larger than that of Cu^2+^ (32.5%), indicating that the Cu metal component is successfully reduced to the obtained composite material, as shown in Figure 4c [44]. Here, there is a small amount of Cu^2+^ present, probably because the Cu^2+^ added during the experiment is excessive.

### 3.2. Catalytic Performance of Pd-Cu Nanocomposites

**Figure 5:**
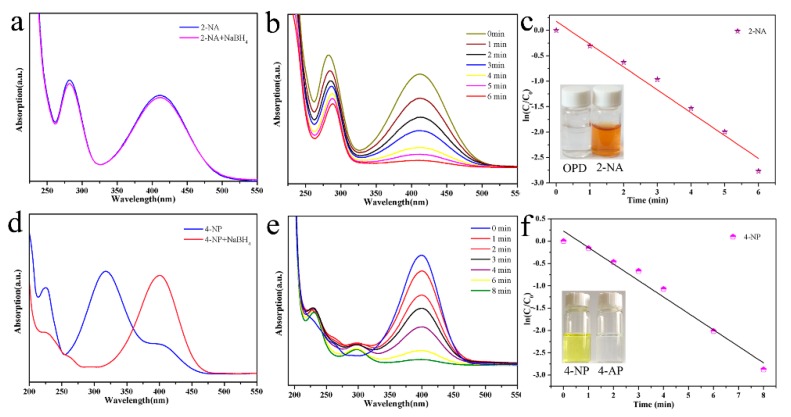
Catalytic reduction test of 2-NA transforming to o-phenylenediamine (OPD) (**a**,**b**) and 4-NP transforming to 4-AP (**d**,**e**) by Pd-Cu bimetallic composite materials (BCM) samples; and relationship between ln(C_t_/C_0_) and the reaction time (t) of reduction catalyst (**c**,**f**).

Next, the obtained Pd-Cu bimetallic composite (BCM) was used as a catalyst to study its catalytic performance for the nitro compound (4-NP, 2-NA) [45,46,47,48,49,50,51,52,53]. As shown in Figure 5, the catalytic performance of Pd-Cu bimetallic composite (BCM) for 2-NA reduction was investigated. As shown in Figure 5a, after the addition of fresh NaBH_4_ to the 2-NA solution, the absorption peak of the mixed solution showed no significant change. After adding the Pd-Cu bimetallic composite (BCM) catalyst for 30 min, the 2-NA of the solution was completely reduced to o-phenylenediamine (OPD). Experiments show that the experimentally added Pd-Cu catalyst has a strong catalytic ability for the reduction of 2-NA.

The catalytic activity of the synthesized Pd-Cu bimetallic composite (BCM) was evaluated by catalytic reduction of p-nitrophenol with a NaBH_4_ solution. As shown in Figure 5d, when NaBH_4_ was not added, the 4-NP solution was pale yellow and the absorption peak was 317 nm. After the addition, the solution changed from light yellow to bright yellow due to the large amount of p-nitrophenol ions, and the absorption peak shifted to 400 nm. The absorption peak of 4-AP then appeared near 295 nm, at which time the solution became colorless. The pseudo-first order equation of kinetics can be used to assess the rate of catalytic reduction reactions. The absorbance of 2-NA is proportional to the concentration of the solution, so we can describe the catalytic reduction by a linear relationship between ln(C_t_/C_0_) (C_t_ represents the concentration of the mixed solution at time t, and C_0_ represents the initial concentration) And the change law of reaction time t. The kinetics of the reduction reaction is shown in Figure 5f, and its linear relationship is consistent with the first kinetic equation. The reaction rate constant k is fitted to the pseudo-level k of O-nitrophenol catalyzed by Pd-Cu bimetallic composite (BCM), and the calculated value is 0.015 s^−1^. In addition, as a comparison, the reaction rate constant k (0.069 s^−1^) fits the pseudo first-order reaction of Pd-Cu bimetallic composite (BCM) catalyst to p-nitrophenol. Obviously, Pd-Cu bimetallic composite (BCM) has a better catalytic effect on 2-NA.

**Figure 6:**
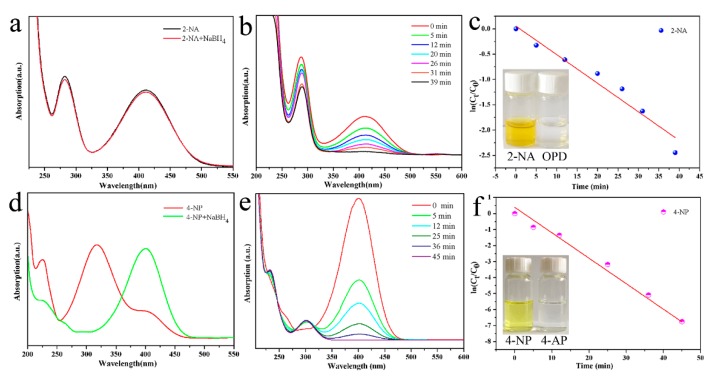
Catalytic reduction test of 2-NA transforming to OPD (**a**,**b**) and4-NP transforming to 4-AP (**d**,**e**) 2-NA,4-NP catalytic comparison experiment by pure palladium; and relationship between ln(C_t_/C_0_) and the reaction time (t) of reduction catalyst (**c**,**f**).

In order to compare the superiority of the Pd-Cu bimetallic composite catalyst, a series of comparative tests were conducted. Figure 6 is a graph of the results of the catalyzing 2-NA and 4-NP by pure palladium nanocrystals, prepared by the same seed-mediated method. It can be seen from Figure 6a that the pure palladium nanocrystal catalyzed the completion of the 2-NA catalytic reduction reaction for 39 min, which showed relatively weak results compared with the Pd-Cu bimetallic composite catalyst catalyzing 2-NA. Similarly, the completion time of the 4-NP reduction reaction catalyzed by pure palladium nanocrystals was 45 min, which showed relatively weak results compared with the Pd-Cu bimetallic composite catalyst catalyzing 4-NP. Therefore, it can be concluded that the Pd-Cu bimetallic composite catalyst has excellent catalytic effects on 2-NA and 4-NP.

**Figure 7:**
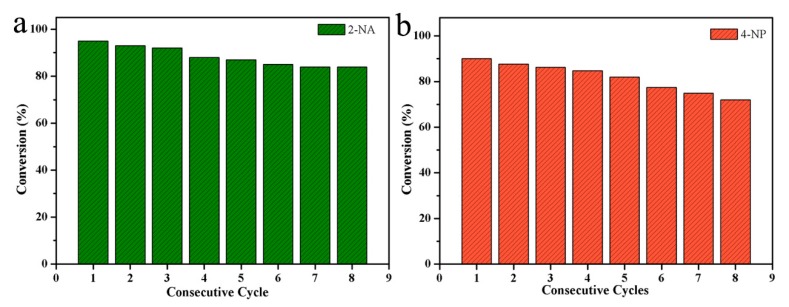
The cyclic catalytic capacity of Pd-Cu bimetallic composite materials (BCM) composite for the reduction of 2-NA (**a**), 4-NP (**b**).

In addition, the widely-used catalyst should have good recyclability and excellent stability. To this end, eight consecutive catalytic experiments were repeated using freshly prepared 4-NP and 2-NA solutions, as shown in Figure 7. The results showed that the stability of the catalyzed 2-NA and 4-NP remained at 84%, and 72%, respectively, after eight cycles. Therefore, the synthesized Pd-Cu bimetallic composite (BCM) has excellent catalytic and stability for catalyzing 2-NA and 4-NP. The catalytic efficiency is slightly reduced due to the loss of nanoparticles during the washing process. In view of the above results, the new Pd-Cu bimetallic composite (BCM) provides analysis for new catalyst fields, such as dye catalytic degradation and sewage treatment, and opens up a new path for the research of palladium series materials and self-assembled composites [54,55,56,57,58,59,60,61,62,63,64].

## 4. Conclusions

In summary, in this work, a homogeneous bead chain Pd-Cu bimetallic composite (BCM) catalyst was proposed to prepare by a seed-mediated method. In particular, the morphology of the bimetallic catalyst can be significantly controlled by sodium dodecyl benzene sulfonate blocking agent and halogen element iodine. The pre-pressed Pd-Cu bimetallic composite (BCM) catalyst exhibits excellent reducing power for the nitro compounds 2-NA and 4-NP. In addition, after eight consecutive cycles of the catalyst, the experimental results showed that the conversion rate of the catalytic 2-NA is still as high as 84%. Interestingly, the bimetallic catalyst showed high catalytic activity and stability. Therefore, this study provides a new method for the sustainable study of new precious metal-transition metal bimetallic catalysts.
